# Plasticity and Tumor Microenvironment in Pancreatic Cancer: Genetic, Metabolic, and Immune Perspectives

**DOI:** 10.3390/cancers16234094

**Published:** 2024-12-06

**Authors:** Ari Hashimoto, Shigeru Hashimoto

**Affiliations:** 1 Department of Molecular Biology, Graduate School of Medicine, Hokkaido University, Sapporo 060-8638, Japan; 2 Division of Molecular Psychoimmunology, Institute for Genetic Medicine, Hokkaido University, Sapporo 060-0818, Japan

**Keywords:** PDAC, tumor microenvironment (TME), metabolic reprogramming, ARF6, Arid5a

## Abstract

Cancer has long been believed to be a genetic disease caused by the accumulation of mutations in key genes involved in cellular processes. However, recent advances in sequencing technology have demonstrated that cells with cancer driver mutations are also present in normal tissues in response to aging, environmental damage, and chronic inflammation, suggesting that not only intrinsic factors within cancer cells, but also environmental alterations are important key factors in cancer development and progression. Pancreatic cancer tissue is mostly comprised of stromal cells and immune cells. The desmoplasmic microenvironment characteristic of pancreatic cancer is hypoxic and hypotrophic. Pancreatic cancer cells may adapt to this environment by rewiring their metabolism through epigenomic changes, enhancing intrinsic plasticity, creating an acidic and immunosuppressive tumor microenvironment, and inducing noncancerous cells to become tumor-promoting. In addition, pancreatic cancer has often metastasized to local and distant sites by the time of diagnosis, suggesting that a similar mechanism is operating from the precancerous stage. Here, we review key recent findings on how pancreatic cancers acquire plasticity, undergo metabolic reprogramming, and promote immunosuppressive microenvironment formation during their evolution. Furthermore, we present the following two signaling pathways that we have identified: one based on the small G-protein ARF6 driven by *KRAS/TP53* mutations, and the other based on the RNA-binding protein Arid5a mediated by inflammatory cytokines, which promote both metabolic reprogramming and immune evasion in pancreatic cancer. Finally, the striking diversity among pancreatic cancers in the relative importance of mutational burden and the tumor microenvironment, their clinical relevance, and the potential for novel therapeutic strategies will be discussed.

## Simple Summary

Cancer initiation and progression are controlled not only by intrinsic factors, such as genetic mutations and mesenchymal trait acquisition, but also by dynamic spatiotemporal shifts within the tumor microenvironment. These elements induce plasticity, and enhance invasiveness, metastasis, and treatment resistance, while promoting immune evasion through the reprogramming of the immune microenvironment. However, the molecular mechanisms driving this reprogramming remain unclear. In this review, we summarize the recent pivotal findings on pancreatic cancer, which is a highly malignant disease, and overview the roles of plasticity, metabolic reprogramming, and the creation of an immunosuppressive microenvironment in disease progression. We also present our research on two crucial signaling pathways, i.e., the dynamic interplay between mutational load and the tumor microenvironment, and the acquisition of cancer plasticity via inflammatory responses and metabolic reprogramming. A comprehensive understanding of these processes is expected to transform therapeutic strategies and improve the outcomes of pancreatic cancer patients.

## 1. Introduction

Nearly 50 years ago, Nowell’s studies on chronic myelogenous leukemia led to the discovery of the Philadelphia chromosome, which was the first cytogenetic abnormality in cancer to be identified [1]. Nowell proposed that cancer develops from a few genetic mutations, evolving into a heterogeneous clonal population dominated by malignant subclones adapted within the tumor tissue, similar to Darwinian evolution [1]. This concept has been widely accepted in cancer biology. Recent sequencing technologies have demonstrated that cancer tissues are composed of cell clusters with highly complex and heterogeneous somatic mutations, clarifying the clonal structure and evolutionary history of cancer [2,3,4,5,6]. Interestingly, genomic analyses have shown that clonal expansion with cancerous driver mutations frequently occurs in phenotypically normal and noncancerous tissues [7,8,9,10,11,12], suggesting that tumorigenesis progresses through the interplay between precancerous and noncancerous cells in the tumor microenvironment (TME), while being affected by aging, inflammation, and other environmental factors.

In the model of tumorigenesis progression through the Darwinian process of evolution, it is assumed that mutations are acquired progressively and sequentially over time. However, several lines of evidence suggest that large numbers of genomic aberrations, such as chromosomal instability, chromoplexy, and chromothripsis, may occur in cancer cells during a short period [13,14,15,16]. In contrast, Motoo Kimura proposed the neutral mutation–random drift hypothesis of molecular evolution and polymorphism, which states that most mutational substitutions detected by comparative studies of DNA sequences are the result of the random fixation of selectively neutral or nearly neutral mutations [17]. According to this hypothesis, genetic mutations resulting from aging or changes in the external environment can be interpreted as the selection and clonal accumulation of cancer-promoting mutations prior to tumorigenesis, which has been tested in a few cancer types [18,19]. These genetic mutations are sufficient for tumor formation and development, and changes that occur during cancer progression contribute minimally. However, it has been pointed out that, whereas untreated tumors may undergo neutral evolution during tumor progression, the emergence of strong selective pressures, such as microenvironmental changes, metastasis, and therapeutic intervention, may select and expand previously neutral mutations [19,20].

The idea that cancer is not merely an autonomous or genetic problem has been highlighted in detail by Mina Bissell and colleagues. Specifically, chickens infected with the oncogenic Rous sarcoma virus exhibited no discernable phenotype until they were subjected to wound formation or application of tumorigenic agents, which subsequently triggered tumor development [21]. This implies that the development of tumors in cells with oncogenic mutations depends on environmental factors, and is affected by additional cell–cell interactions, such as the tissue microenvironment. Changes in various components of the TME over time, and in the characteristics of plastically transformed cells as they become malignant and metastatic, have been observed [22,23,24]. Moreover, it has been demonstrated that numerous nongenetic changes occur in tumors, such as alterations in epigenetic transcriptional regulation through chemical modifications of histones and DNA [25]. Compared with normal tissues, the noncancerous cell population in the TME is complex, consisting of a diverse network of cells including immune cells, fibroblasts, blood vessels, neurons, and adipocytes. Cancer-originating cells, which are responsible for self-renewal, maintenance, proliferation, metabolic reprogramming, invasion, and metastasis, integrate various signals from the TME and generate new signals to initiate and promote tumorigenesis, demonstrating high plasticity [26,27,28].

Pancreatic ductal adenocarcinoma (PDAC), which accounts for about 90% of pancreatic cancers, has the highest mortality rate of all cancers, with a 5-year survival rate of patients of only about 10% in the United States [29]. Although the exact cause of cancer death is debated [30], metastasis greatly contributes to the high mortality rate. At the time of diagnosis, only 20% of PDACs are still localized in the pancreatic tissue, approximately 30% have spread to regional lymph nodes, and more than 50% have metastasized to the liver and lungs [31]. Early diagnostic methods are lacking owing to the absence of or vague symptoms, insufficient early-stage diagnostic biomarkers, and the anatomy of the pancreas making it difficult for regular access and diagnosis. Current therapies for PDAC include surgery, chemotherapy (such as with gemcitabine and FOLFIRINOX, comprising fluorouracil, leucovorin, irinotecan, and oxaliplatin), and radiation therapy. The efficacy of these therapies is marginal, and only slightly prolongs the survival of PDAC patients [32,33]. Immunotherapies targeting immune checkpoint molecules, such as cytotoxic T-lymphocyte antigen-4 (CTLA-4), programmed death-1 (PD-1), or their ligands, have had some success in treating several types of cancers, but not in treating PDAC. To date, clinical trials for PDAC have shown no success, even in trials testing combinations of multiple immune checkpoint inhibitors or combinations of immune checkpoint inhibition and chemotherapy [34,35,36]. In recent years, owing to factors such as obesity and increasing life expectancy, the incidence of PDAC has been increasing steadily [37], and it is estimated that the disease will become the leading cause of morbidity by about 2030 [38]. Thus, there is an urgent need to identify and develop new diagnostic and therapeutic approaches to this serious disease.

In PDAC, mutations in four major driver genes have been identified, namely, *KRAS*, *TP53*, *CDKN2A*, and *SMAD4* [39,40,41]. Constitutively activating mutations in *KRAS*, which often demonstrate oncogenic activity, are found in more than 90% of patients, and are thought to be implicated in the early stages of neoplastic formation in PDAC [41,42,43,44]. Additionally, oncogenic *KRAS* mutations have been demonstrated to promote tumor proliferation and survivability by enhancing tumor signaling via metabolic reprogramming and intercommunication with stromal cells [45,46]. Recently, inhibitors of specific oncogenic *KRAS* mutations have been developed, and hold promise as a new therapeutic strategy against PDAC [47,48,49,50,51]. On the other hand, recent genomic analyses have identified low-frequency (<10%) genetic mutations that are associated with specific subsets of PDAC [40,52,53]. Interestingly, these mutations have been shown to occur in a set of genes involved in DNA stability, DNA repair, epigenetic regulation, and neuroaxonal guidance. Notably, the burden of genetic mutations in PDAC is known to be limited compared with many common solid tumors, such as melanoma, non-small-cell lung cancer, and bladder cancer [54,55]. Taken together, these findings suggest that nongenetic factors contribute to the malignancy of PDAC.

A hallmark of PDAC is that nonmalignant tumor components comprise the majority of the TME. The TME includes myofibroblasts, inflammatory fibroblasts, endothelial cells, pericytes, and immune cells, all surrounded by a dense and complicated extracellular matrix (ECM) [56]. Thus, TMEs in PDAC demonstrate poor nutrient status, hypoxia, oxidative stress, inflammatory stress, extracellular acidosis, and reduced angiogenesis [33,57,58]. These harsh conditions create strong selection pressures, resulting in the existence of only specific cell populations, including both cancerous and noncancerous cells, that can adapt their metabolism to survive and proliferate. Notably, these adaptive capacities in the PDAC TME are linked to increased invasiveness, metastatic potential, stem cell-like properties, and therapeutic resistance in PDAC cells [59]. Supporting this, several genomewide gene expression profiling and genome sequencing studies aimed at understanding the comprehensive molecular landscape of PDAC have demonstrated the presence of a basal-like (also known as quasimesenchymal or squamous-like) subtype, which is associated with a less favorable prognosis than the other subtypes [52,53,60,61], immunologically ‘cold’ tumors, and being resistant to immunotherapy [62]. Moreover, metabolite profiling and transcriptome analysis of PDAC have shown that the basal-like subtype is associated with the glycolytic subtype [63,64], suggesting that metabolic reprogramming plays a crucial role in tumor progression, and is expected to lead to new therapeutic strategies. However, the plasticity of PDAC cells, owing to intrinsic heterogeneity in gene expression and metabolic characteristics, as well as immunopathological changes linked to physicochemical and biological alterations in the TME, render PDAC a particularly challenging disease to cure [63,64,65].

The association between the nervous system and cancer represents an exciting frontier in biomedicine that is not addressed in this review. There are accumulating lines of experimental evidence indicating various aspects of this association, ranging from the systemic effects of cancers on neurological function, such as cachexia, cognitive impairment, and sleep disturbances, to the regional remodeling of tissue dominance by tumors and the regulatory effects of the nervous system on tumor phenotype. Recent review articles are available on these topics [66,67,68,69]. Furthermore, although there is little direct evidence that neurological stress affects tumor initiation and progression, as evidenced by epidemiological studies and mouse models, stress has been shown to affect tumor progression, dissemination, and therapeutic outcomes [70]. Although many research groups have started to analyze neuroimmune communication within tumors, it still remains unclear as to whether stress affects local neural remodeling, and immune and metabolic regulation within the TME associated with PDAC plasticity.

In this review, we highlight the recent discoveries on how pancreatic cancer cells develop and enhance mesenchymal plasticity through metabolic and immune reprogramming during tumor progression. We also explore how mesenchymal tumors orchestrate metabolic network rewiring to create an immune-invasive TME. Additionally, we present two signaling pathways, namely, the small G-protein ADP-ribosylation factor 6 (ARF6)-based signaling pathway driven by *KRAS/TP53* mutations, and the RNA-binding protein AT-rich interactive domain-containing protein 5A (ARID5A)-based signaling pathway mediated by inflammatory cytokines, which promote PDAC plasticity via metabolic reprogramming and immune evasion. Finally, using PDAC as a representative cancer model, we discuss the diverse effects of genomic mutations and the surrounding tumor environment, their clinical relevance, and research strategies to develop novel diagnostic and therapeutic approaches for PDAC.

## 2. PDAC Initiation and Heterogeneity

### 2.1. Origin of Pancreatic Cancer

The adult pancreas is composed of two functionally and morphologically distinct components, i.e., exocrine and endocrine tissues. The exocrine pancreas accounts for more than 95% of total pancreatic tissue, and is composed of acinar cells that produce digestive enzymes and ductal cells that deliver these enzymes to the intestine. Interestingly, the mammalian pancreas can regenerate after injury even in adults, and, in humans, acinar cells are responsible for plasticity. For example, analyses using mouse models have shown that acinar cells can dedifferentiate into a progenitor cell-like state with a tubular appearance. This phenomenon has been termed acinar-to-ductal-metaplasia (ADM) [71,72]. In addition, ADM occurs via the activation of nuclear factor-κB (NF-κB) in chronic pancreatitis, and has been associated with the formation of precancerous lesions called pancreatic intraepithelial neoplasia (PanIN) [73,74,75]. Thus, acinar cells of pancreatic exocrine tissue maintain plasticity to adapt to changes in the external environment, and it is speculated that the dysregulation of this plasticity is associated with the development of pancreatitis and pancreatic cancer.

In the progression to malignant carcinoma, in which invasive and metastatic traits are acquired by the pancreas, three precancerous pathologies with different characteristics occur. PanIN is the most frequently observed precursor lesion, and is the most frequently observed metastatic lesion [76]. Intraductal papillary mucinous neoplasms (IPMNs) and mucinous cyst neoplasms (MCNs), on the other hand, are also recognized as precursor lesions of invasive pancreatic cancer, with IPMNs originating from the main pancreatic duct [77]. Approximately 80% to 85% of PDACs occur in the setting of PanIN lesions, whereas the remaining 15% to 20% are associated with IPMN and MCN lesions [77,78,79,80].

### 2.2. Genomic Alterations in Pancreatic Cancer

As mentioned above, although the mutational burden of PDAC is low compared with other solid tumor types [54,55], mutations in four major driver genes, namely, *KRAS*, *TP53*, *CDKN2A*, and *SMAD4* [39,40,41], have also been identified, and the multistep accumulation of mutations during tumor progression have been investigated in detail [39,40,52,53]. *KRAS* mutations are observed in most PanINs and IPMNs, suggesting that oncogenic *KRAS* mutations are an early event in PDAC tumorigenesis [81,82,83,84]. The small guanosine triphosphatase (GTPase) KRAS activates mitogen-activated protein kinase, also known as extracellular signal-regulated kinase (MAPK-ERK) signaling, and has physiological functions in cell proliferation, differentiation, migration, and survival [84]. On the other hand, oncogenic *KRAS* mutations have been shown to be involved in the development of pancreatic cancer from chronic pancreatitis [85,86,87], indicating that enhanced MAPK-ERK signaling is involved in promoting pancreatic cancer progenitor cell expansion and tumorigenesis by stromal cell populations within the inflammatory TME. Furthermore, *KRAS* mutations protect against inflammation-associated senescence [88,89], and also improves cell fitness and plasticity via promoting autophagy, micropinocytosis, and the formation of stress granules [90,91,92].

Interestingly, approximately 10% of early-onset PDACs do not have constitutively activating mutations in *KRAS*. Mutations in genes such as *neuregulin-1*, *ret proto-oncogene*, *B-Raf proto-oncogene*, *serine/threonine kinase*, *ALK receptor tyrosine kinase*, *neurotrophic receptor tyrosine kinase 1*, and *epidermal growth factor receptor* (*EGFR*) are found in these cases, and these mutations have been shown to selectively activate MAPK-ERK signaling, which is a transduction pathway downstream of *KRAS* signaling [93].

In addition to *KRAS* mutations, the PDAC genomic sequence has a large number of loss-of-function mutations in *CDKN2A*, *SMAD4*, and *TP53* [39,40,52,53]. The sequential appearance of these alterations during the multistep progression of PDAC suggests that *TP53* and *SMAD4* mutations usually occur late in pancreatic tumorigenesis, in both PanIN and IPMN [94,95].

*CDKN2A* mutations often involve homozygous deletions at the chromosome 9p21 locus, and are associated with the codeletion of genomically adjacent interferon clusters in about half of the patients with PDAC. The deletion of interferon clusters has been reported to induce an immunologically “cold” TME, and resistance to immunotherapy [96]. SMAD4 is a tumor suppressor and a key regulator of the transforming growth factor (TGF)-β signaling pathway [97]. Loss of SMAD4, which occurs late in pancreatic tumorigenesis, promotes cancer cell proliferation and motility by the dysregulation of the TGF-β pathway, causing tumor chemoresistance, invasion and metastasis, immune evasion, etc. [98]. Genomic mutations in *TP53* are found in approximately 80% of PDACs, and most of them are missense mutations that result in the acquisition of novel functions, such as DNA binding and aberrant molecular interactions [99,100]. TP53 mutations are associated with increased genomic instability [101], the reprogramming of the cellular metabolism [102,103], and the acquisition of metastatic traits [104].

Although it is not discussed in this review, genetic variants that are less frequent than mutations in major driver genes associated with DNA maintenance and the damage response, such as epigenetic chemical modifications, have been identified and shown to be associated with the phenotypic diversity of PDAC [105,106,107].

### 2.3. Transcriptional Subtype Classification

Transcriptional processes are the primary determinant of gene expression, and have major effects on cellular and tissue phenotypes. In pancreatic cancer, rather than genomic mutations, the comprehensive and extensive analysis of mRNA expression levels has led to the classification of PDAC into subtypes, which are associated with patient prognosis. First, Collisson et al. analyzed both patient microarray datasets and PDAC cell lines to classify pancreatic cancer subtypes into three groups (i.e., “classical”, “quasi-mesenchymal”, and “exocrine-like”) [60]. Importantly, they showed that “quasi-mesenchymal” cell lines are sensitive to gemcitabine, and that erlotinib is effective against the “classical” subtype, potentially predicting drug responses in these subtypes. Bailey et al. then used RNA sequencing (RNA-seq) methods to classify PDAC into four groups (“squamous epithelial”, “pancreatic progenitor cell”, “immunogenic”, and “abnormally differentiated endocrine–exocrine [ADEX]” subtypes) [52]. However, this subtyping was problematic because the majority of PDAC tumor tissue consists of stromal cell populations, including immune cells [40,52]. To overcome this problem, Moffit performed “virtual microdissection”, which statistically subtracted the transcription background owing to stromal and immune cells from the bulk RNA-seq signatures [61]. This enabled them to classify the stromal cell population into two subtypes, namely, “normal stroma” and “activated stroma”, and they proposed that the PDAC tumor cell population could be divided into two subtypes, namely, “classical” and “basal-like”.

On the other hand, Puleo et al. used the laser capture microdissection (LCM) method to harvest epithelial or stromal-rich tissue areas, to overcome the issue that the majority of PDAC TMEs are stromal cell populations, and classified them into five groups (i.e., “pure basal-like”, “stromal activated”, “desmoplastic”, “pure classical”, and “immunoclassical”) [108]. This analysis demonstrated an overlap with the Moffitt and Bailey subtypes, suggesting that the Bailey ADEX subtype is likely a result of the contamination of pancreatic acinar cells.

Similarly, Maurer et al. found that isolated epithelial tissue mainly comprised the Moffitt classical or basal-like subtype, but epithelium-rich tumor samples mainly comprised the Bailey “ADEX” or Collisson “exocrine-like” subtype, and found no lines of evidence for the existence of an “exocrine-like” subtype [109]. The lack of ADEX or exocrine-like subtypes found in these two studies is consistent with the observations of The Cancer Genome Atlas. That is, ADEX or exocrine-like signatures in samples are associated with a low PDAC content [53], suggesting that these signals originate from stromal, immune, or noncancerous elements of the tissue. Finally, the Maurer stromal region of PDAC tumors were hypothesized to be comprised of two prominent subtypes, namely, the “ECM-rich” and “immune-rich” subtypes [108].

To overcome the limitations of these studies, Chan-Seng-Yue et al. introduced the single-cell RNA-seq (scRNA-seq) method, which can identify cells within the TME by their gene expression patterns. They used LCM-enriched epithelial tissue from patients with advanced PDAC and identified the following five different subtypes: basal-like-A, basal-like-B, hybrid, classic-A, and classic-B [5]. Importantly, we have shown that different subtypes can coexist within individual pancreatic tumors.

Based on previous analyses, the subtyping of PDACs by their mRNA expression levels is still problematic, although a consensus is forming for the two major subtypes, i.e., the classic and basal-like subtypes [110]. This may be owing to the plasticity caused by the variation in transcriptional responses in PDAC cells in adapting to changes in biological influences, such as humoral factors and metabolites generated by immune cells and stromal cell populations, which constitute the bulk of the TME, as well as physicochemical effects, such as mechanical stress and hypoxia [111].

## 3. PDAC Plasticity and Metastasis

Metastasis is a major cause of death from cancer. As mentioned above, distant metastasis is present in approximately 50% or more of PDAC patients at the time of diagnosis [31]. Why do pancreatic cancers metastasize early?

In metastasis, cancer cells undergo a diverse series of biological events, including local invasion, extravasation into blood vessels, floating in the bloodstream, intravasation into target tissues, dormancy, and development. Cells of metastatic origin (MOCs) need to acquire invasive and metastatic abilities within the primary tumor, and eventually clear many obstacles in the metastasis-forming process [112]. From the perspective of invasion-specific and metastasis-specific genetic mutations, a recent study investigating the association between genomic alterations and the metastasis of 50 different types of tumors in more than 25,000 patients with metastatic cancer showed that *TP53* mutations, the deletion of *CDKN2A*, and the amplification of *MYC* were more frequently altered in metastatic pancreatic tumors [113]. In addition, increased genomic complexity, presumably owing to TP53 mutations, was observed in metastatic samples from 16 out of the 50 cancer types, with the gain of chromosome 12p, where *KRAS* is located, being particularly frequent in pancreatic cancer. Furthermore, notably, it was suggested that, at the level of driver mutations, the selection pressures acting in primary and metastatic tumors are often similar [113].

On the other hand, it is becoming clear that cancer cell plasticity owing to intrinsic nongenetic factors is a driving force for the acquisition of invasive and metastatic traits [114,115,116]. Cancer cell plasticity may increase the frequency of MOC phenotypes owing to the accumulation of diverse genomic variants in cancer cell populations, expanding the phenotypic complexity. Moreover, plasticity is dynamic and reversible, and can induce traits that adapt phenotypes to the fluctuating microenvironment in the metastatic process at a higher rate than genetic changes that depend on stochasticity [114,115,116]. Cellular plasticity can be elicited by adaptation to metabolic fluctuations, epigenomic modifications, transcriptional rewiring of enhancer and promoter activities, or translational reprogramming. MOCs face complex and rapidly changing TMEs, and, therefore, having a large number of cells with a high capacity to acquire plastic traits as a cell population improves the probability of maintaining a selective advantage. Whereas cancer cells face fluctuating environmental selection pressures, they may also direct the formation of locally more favorable microenvironments [4]. Indeed, a growing number of recent reports support that the TME and MOCs coevolve [23,117,118].

In pancreatic cancer, the availability of oxygen and nutrients is restricted, which thereby promotes cell motility and invasiveness via the epithelial–mesenchymal transition (EMT) [119]. The EMT is a cellular program that often occurs in both normal and pathological processes. The EMT has been reported to be essential for embryonic development and wound healing, as well as being responsible for invasiveness and metastatic potential, drug resistance, the avoidance of cellular senescence, and even stemness in many types of cancer [120,121,122,123]. It has also been suggested that the EMT is also involved in the regulation of antitumor immunity, as it has been shown to increase resistance to elimination by cells of the adaptive immune system that are present in the tumor-associated stroma [122,124].

Recent studies have raised the possibility that the involvement of a complete EMT program may actually reduce metastatic tendencies [125,126]. Single-cell analysis combined with lineage tracing highlighted that the EMT involved in cancer metastasis is not a binary switch between epithelial and mesenchymal lineages, but includes hybrid states that transiently show both epithelial and mesenchymal traits [127,128]. Importantly, cells with hybrid EMT traits are more efficient at forming colonies in distant organs than mesenchymal cells expressing the full EMT program [127,128]. In particular, invasion and metastasis were shown to be promoted by rare subclones with hybrid EMT characteristics in a mouse model of pancreatic cancer [129,130]. These findings suggest that the phenotype demonstrated by hybrid EMT cancer cells is highly plastic, capable of transiently acquiring either epithelial or mesenchymal traits depending on the external environment. This strongly suggests that hybrid EMT cancer cells have the plasticity required for MOCs, in particular the ability to reprogram epitheliality, including proliferative ability, at distant metastatic sites.

## 4. Metabolic Reprogramming in Pancreatic Cancer

PDAC has a limited supply of oxygen and nutrients from the blood, owing to the desmoplastic barrier characteristic of its TME. However, PDAC demonstrates various adaptations to survive under such harsh conditions, meeting the energy demands required for proliferation and even for acquiring invasive and metastatic abilities. Major mechanisms for this include intrinsic factors of PDAC, such as genetic mutations (particularly the metabolic reprogramming of glucose, glutamine, and lipids mediated by oncogenic *KRAS*), and the recycling and scavenging of intracellular and extracellular nutrients through autophagy and micropinocytosis [84]. Additionally, metabolic heterogeneity resulting from the diversity of gene expression in PDAC has been demonstrated. Furthermore, extrinsic factors, including nutrients supplied from stromal cells, such as fibroblasts and macrophages, accumulate in the TME.

### 4.1. Glucose Metabolism

Glucose is a vital carbon source for cell growth and survival, which is catabolized through glycolysis and the tricarboxylic acid (TCA) cycle. These pathways produce adenosine triphosphate (ATP) and generate carbon intermediates that are essential for macromolecular biosynthesis. Oncogenic *KRAS* mutations in PDAC enhance the expression of key metabolic enzymes associated with the glucose metabolism, including those involved in glycolysis, hexosamine biosynthesis, and the pentose phosphate pathway (PPP) [45]. These mutations also increase glucose consumption by upregulating glucose transporter 1 (GLUT1), hexokinase 1 and 2 (HK1 and HK2), and lactate dehydrogenase A (LDHA) [131].

In the hypoxic TME of PDAC, the transcription factor hypoxia-inducible factor 1α (HIF-1α) drives metabolic reprogramming, increasing the expression of glycolytic enzymes and lactate production. Stabilized HIF-1α induces GLUT1 expression, enhancing glucose uptake and supporting aerobic glycolysis [132,133,134]. HIF-1α also upregulates LDHA and monocarboxylate transporter 4 (MCT4), promoting a shift from oxidative phosphorylation (OXPHOS) to glycolysis, and thus maintaining ATP production under low-oxygen conditions [135,136,137]. MCT4 exports lactate from the cell, aiding in this metabolic shift. Therefore, HIF-1α promotes the metabolic shift from oxidative phosphorylation to glycolysis in hypoxic conditions. This is not only beneficial for maintaining the homeostasis of biological energy, but may also contribute to tumor survival and growth.

**Figure 1:**
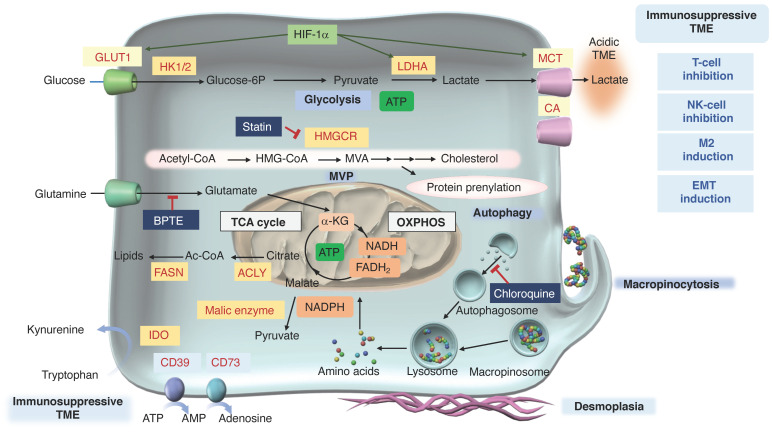
Metabolic reprogramming in cancer. The dense fibrotic barrier characteristic of PDAC limits the supply of oxygen and nutrients from the bloodstream. However, PDAC cells adapt to this harsh environment by not only securing the energy needed for survival, but by also acquiring the ability to invade and metastasize. Key mechanisms include the reprogramming of glucose, glutamine, and the lipid metabolism driven by oncogenic KRAS, as well as nutrient recycling through autophagy and macropinocytosis. Oncogenic KRAS mutations in PDAC cells upregulate GLUT1, HK1, HK2, and LDHA, leading to increased glucose consumption. When glycolysis is upregulated in tumor cells, lactate production increases, leading to the formation of an acidic TME. This promotes mesenchymal traits and supports an immunosuppressive TME. In the hypoxic TME, HIF-1α activates GLUT1, enhancing glucose uptake, and upregulates LDHA and MCT4, promoting a shift from oxidative phosphorylation (OXPHOS) to glycolysis. This shift enables ATP production to be maintained under hypoxic conditions, whereas MCT4 facilitates the export of lactate, supporting the metabolic reprogramming. Glutamine is essential for ATP synthesis through the TCA cycle and OXPHOS, playing a crucial role in tumor growth. The glutaminase inhibitor BPTES substantially inhibits the proliferation of PDAC. The MVP is an essential lipid metabolic pathway required for cholesterol biosynthesis and the prenylation of proteins. HMGCR inhibitors, which inhibit the rate-limiting step of the MVP, such as statins, are used to lower cholesterol levels and are also being investigated as anticancer agents. The upregulation of autophagy is a characteristic of PDAC, and the autophagy inhibitor chloroquine suppresses tumor proliferation. Macropinocytosis actively produces free amino acids that support tumor metabolism and occurs in PDAC cells with *KRAS* mutations. IDO, which converts tryptophan to kynurenine, is often overexpressed in PDAC. The depletion of tryptophan and the production of kynurenine in the TME create an immunosuppressive environment that weakens antitumor T-cell responses. ATP has immunostimulatory effects; however, it is converted to AMP by the ectonucleotide triphosphate diphosphohydrolase CD39, and then AMP is converted to adenosine by CD73. CD73 plays a crucial role in regulating the immunosuppressive microenvironment in PDAC and may promote tumor progression. HK1/2, hexokinase1/2; LDHA, lactate dehydrogenase A; GLUT, glucose transporter; MCT, monocarboxylate transporter; CA, carbonic anhydrase; HMGCR, HMG-CoA reductase, MVA, mevalonic acid; MVP, mevalonate pathway; α-KG, α-ketoglutarate; FASN, fatty acid synthase; ACLY, ATP-citrate lyase; NK, natural killer; M2, M2 macrophage.

Increased glycolysis in tumor cells leads to high lactate production, creating an acidic TME. Excess lactate inhibits CD8^+^ T-cells and natural killer (NK) cells [138], whereas it promotes immunosuppressive cells, such as M2 macrophages [139]. Tumor cells adapt the acidic microenvironment by regulating the pH using monocarboxylate transporters and carbonic anhydrases [140]. The exposure of cells to lactic acidosis induces an EMT phenotype and enhances interleukin (IL)-8 expression, which contributes to tumor angiogenesis and metastasis by using lactate as an energy source [141,142]. High lactate levels in PDAC are associated with an unfavorable disease prognosis [143], suggesting that the acidic tumor environment leads to the acquisition of mesenchymal traits and an immunosuppressive TME (Figure 1).

### 4.2. Glutamine Metabolism

Glutamine acts as a mitochondrial substrate for synthesizing macromolecules in cancer cells, providing carbon for the TCA cycle and acting as a major nitrogen donor for nucleotide and amino acid production [144]. “Glutamine addiction” is common in PDAC [145,146,147]. In mitochondria, glutamine is crucial for ATP synthesis via the TCA cycle and OXPHOS, which is essential for tumor growth [148,149]. Glutamine is the most abundant nonessential amino acid in the blood and plays various metabolic roles [146,150].

Glutamine is converted to glutamate by glutaminase, and then to α-ketoglutarate (α-KG) by glutamate dehydrogenase in mitochondria. α-KG enters the TCA cycle, supplying intermediates such as citrate and malate, and generating nicotinamide adenine dinucleotide and flavin adenine dinucleotide for ATP production. Glutamine also generates nicotinamide adenine dinucleotide phosphate (NADPH) through glutaminolysis, in which malate is converted to pyruvate by malic enzyme. Mitochondrial isocitrate dehydrogenase (IDH) 2 reductively carboxylates glutamine-derived α-KG to isocitrate, which is then isomerized to citrate. Citrate transported to the cytoplasm is reversibly converted to isocitrate by aconitase, and then to α-KG by cytoplasmic IDH1, producing NADPH for lipid synthesis. PDAC has been shown to metabolize glutamine to maintain NADPH levels and cellular redox homeostasis, which are essential for proliferation [145].

Circulating glutamine is taken up through transporters, such as solute carrier family 1 member 5 (SLC1A5, also known as alanine–serine–cysteine transporter 2), and exchanged for branched-chain amino acids (BCAAs), including leucine, isoleucine, and valine, by solute carrier family 7 member 5 (SLC7A5, also known as L-type amino acid transporter 1). In the cytoplasm, BCAAs are broken down by branched-chain amino acid transaminase (BCAT) 1, and, in mitochondria, by BCAT2, producing branched-chain α-ketoacids and glutamate. Early pancreatic cancer driven by mutant *KRAS* shows increased plasma BCAAs, with BCAT2 highly expressed in ductal cells [151]. The BCAA-BCAT2 axis driven by KRAS is important in PDAC development [152]. The overexpression of amino acid transporters (SLC1A5 and SLC7A5) is associated with an unfavorable disease prognosis [153].

PDAC cells are highly dependent on glutamine for their growth [145,154]. The glutaminase inhibitor BPTES (Bis-2-(5-phenylacetamido-1,2,4-thiadiazol-2-yl)ethyl sulfide) significantly inhibits PDAC growth, but does not affect cell death. Glutamine depletion activates autophagy, which is associated with macropinocytosis, thereby regulating intracellular glutamine levels. Both glutamine depletion and autophagy inhibition robustly activate apoptosis [155]. However, glutamine depletion also induces invasive and metastatic traits via increased ERK signaling and activating transcription factor 4 (ATF4) activation, thereby upregulating the EMT regulator Slug [156]. Therefore, the simultaneous inhibition of pathways involved in the glutamine metabolism, such as autophagy and EMT, may provide a new therapeutic approach to PDAC.

### 4.3. Lipid Metabolism

Lipids are crucial components of biomolecules, playing key roles in various cellular processes. During lipid synthesis, ATP-citrate lyase (ACLY) converts citrate to acetyl-CoA, which is then converted to malonyl-CoA by acetyl-CoA carboxylase. Fatty acid synthase (FASN) uses these acyl groups in an NADPH-dependent process to generate long-chain saturated fatty acids [157]. Increased levels of lipid-producing enzymes, including ACLY, are common in PDAC [158,159], and inhibiting ACLY suppresses tumor growth [160]. The overexpression of FASN is linked to an unfavorable prognosis, and resistance to radiotherapy and gemcitabine in patients with PDAC [161], suggesting that the genetic and pharmacological inhibition of FASN and other fat-producing enzymes may be a potential therapeutic target for PDAC.

The mevalonate pathway (MVP) is essential for the lipid metabolism, including cholesterol biosynthesis and protein prenylation. Notably, expression levels of pancreatic 3-hydroxy-3-methylglutaryl-CoA reductase (HMGCR), which is the rate-limiting enzyme of the MVP, were upregulated in a mutant *KRAS*-driven mouse PDAC model [162] and patients with PDAC [163]. In addition, HMGCR has been shown to be a target for PDAC inhibition [162,164]. HMGCR inhibitors, such as statins, are used to lower cholesterol and are being investigated as anticancer agents in several cancer types [165] (Figure 1). In PDAC model mice, the level of acetyl-CoA is increased in pancreatic acinar cells, indicating that the utilization of acetyl-CoA in the MVP supports ADM, and that the pancreatic-specific loss of ACLY, which is involved in acetyl-CoA production, inhibits ADM and tumorigenesis [165]. In addition, growth factors promote AKT-ACLY signaling and histone H3 acetylation in PDAC cells, and a combination of the bromodomain inhibitor JQ1 and statins was shown to inhibit both PDAC cell proliferation and tumor growth [166]. This suggests that the utilization of acetyl-CoA in histone acetylation and the MVP promotes the plasticity and proliferation of PDAC, and indicates the possibility of targeting these pathways as therapeutic strategies.

Consistently, a meta-analysis of more than 170,000 pancreatic cancer patients suggested that statin use significantly reduces pancreatic cancer risk [167], although there was no correlation between increased *HMGCR* expression and shorter survival in pancreatic cancer patients [163]. Interestingly, statins were recently identified from a library of 624 Food and Drug Administration-approved inhibitors as agents that significantly inhibit the metastatic potential of PDAC cells both in vitro and in vivo [168]. Notably, statin treatment induces the EMT-like reprogramming of PDAC cells, which enhances the invasiveness and anti-anoikis ability, thereby resulting in metastasis. However, these cells do not induce the hybrid EMT seen in malignant PDAC and the mesenchymal–epithelial transition, which is the process opposite to the EMT, and cannot be sufficiently transformed into epithelial cells during metastases [168]. These results suggest that the forced induction of the mesenchymal phenotype by statins may lead to a new therapeutic approach of suppressing the proliferation at metastatic sites by reducing the plasticity during the metastatic process of PDAC.

### 4.4. Autophagy

Enhanced autophagy is a characteristic of PDAC, involving organelle reprogramming to adapt to the nutrient-limited TME [90,169]. Inhibiting autophagy with genetic or pharmacological inhibitors, such as chloroquine, results in mitochondrial abnormalities, reduced OXPHOS, decreased proliferation in vitro, and tumor growth inhibition in vivo [90]. The importance of autophagy in PDAC tumor formation was further demonstrated by crossing PDAC model mice with conditional knockout mice for the autophagy-essential gene *Atg5* [170,171]. Mitophagy, a selective autophagy of mitochondria driven by *KRAS* mutations, reduces the amount of functional mitochondria, showing its crucial role in redox robustness and tumor formation [172]. Notably, clinical trials have shown that the autophagy inhibitor hydroxychloroquine enhances the response of PDAC patients to chemotherapy with gemcitabine or gemcitabine and nab-paclitaxel [173,174].

As described in Section 5, PDAC is highly immunosuppressive, characterized by the substantial infiltration of myeloid-derived suppressor cells (MDSCs) and a lack of activated cytotoxic CD8^+^ T-cells, making it minimally responsive to immune checkpoint inhibitors (ICIs) such as anti-PD1 and anti-CTLA4 antibodies [62,175,176]. Resistance to ICI therapy is associated with the reduced expression of major histocompatibility complex class I (MHC-I) molecules on PDAC cells, which are mainly localized in the autophagosomes and lysosomes of these cells, and promote immune evasion [177]. Inhibiting autophagy increases the surface levels of MHC-I, which enhances antigen presentation and T-cell responses, and inhibits tumor growth [178]. Combining systemic autophagy inhibition with ICIs demonstrates a synergistic effect, providing a molecular mechanism for autophagy-mediated immune evasion, and supports further research into therapies targeting the autophagy–lysosome pathway in PDAC.

Iron is an essential cofactor for metabolic and cellular processes [179], but free iron can promote cell death through lipid oxidation “ferroptosis” [180]. Intracellular free iron is stored in ferritin to prevent free radical generation [179]. Importantly, autophagy plays a crucial role in maintaining iron homeostasis in PDAC. Ferritin is transported to autophagosomes via nuclear receptor coactivator 4 (NCOA4), and free iron is released through autophagy in a process called “ferritinophagy”. Indeed, increased NCOA4 expression promotes tumor formation, whereas its knockout delays growth [181,182]. Notably, MEK inhibition in PDAC increases lysosome biogenesis and ferritinophagy, supporting the synthesis of iron–sulfur cluster proteins required for respiration [183].

### 4.5. Macropinocytosis

Deprivation of glucose in PDAC was shown to produce reactive oxygen species and induce autophagy to supply the nutrients necessary for proliferation [184]. Glutamine starvation increases macropinocytosis, highlighting its regulatory role in PDAC [185]. Macropinocytosis involves membrane ruffles engulfing extracellular substances, which are degraded in macropinosomes, leading to the production of free amino acids that support the tumor metabolism/anabolism [186] (Figure 1). It is a crucial mechanism for the recycling and scavenging of nutrients, which sustains PDAC growth by taking in extracellular fluid contents [187].

PDAC cells with *KRAS* mutations demonstrate high basal levels of macropinocytosis, consuming extracellular proteins for rapid growth, which is closely associated with autophagy [91,169,188,189,190,191,192]. Autophagy degrades extracellular proteins through macropinocytosis, which supplies amino acids, particularly glutamine, ensuring PDAC cell proliferation [155]. Understanding the dynamic balance between the glutamine metabolism and autophagy associated with macropinocytosis is essential for developing treatments for PDAC. Macropinocytosis could be a therapeutic target, and understanding its coordination with autophagy is crucial for establishing effective treatments.

### 4.6. Other Types of Metabolism

The availability of amino acids, particularly arginine and tryptophan, in the TME is crucial for antitumor immunity. Increased arginine levels are important for T-cell activation, inducing metabolic changes, such as a shift from glycolysis to OXPHOS and promoting memory T-cell differentiation [193]. Indoleamine 2,3-dioxygenase (IDO), which converts tryptophan to kynurenine, is often overexpressed in PDAC [194]. Tryptophan depletion and kynurenine production in the TME create an immunosuppressive environment, weakening the antitumor T-cell response [195] (Figure 1). The mechanistic action of kynurenine is discussed in Section 9.

Extracellular ATP levels can increase significantly under hypoxia [196,197]. ATP has immunostimulatory effects but is ultimately converted into adenosine through a stepwise process. ATP is first converted to adenosine monophosphate (AMP) by the ectonucleotidase CD39 (also known as ectonucleoside triphosphate diphosphohydrolase 1), and then AMP is dephosphorylated by CD73 (also known as 5′-nucleotidase ecto) to adenosine. Adenosine acts on purinergic receptors (A1, A2a, A2b, and A3), which regulate various physiological and pathological processes [198,199,200]. The A2a and A2b receptors are mainly responsible for downstream immunosuppressive signaling associated with intracellular cyclic AMP accumulation [201]. In PDAC, high CD73 expression is associated with an immunosuppressive TME, decreased survival rates, and reduced levels of CD4^+^, CD8^+^, and CD21^+^ tumor-infiltrating lymphocytes (TILs) [202]. Therefore, CD73 plays a crucial role in regulating the immunosuppressive TME in PDAC and may promote tumor progression.

### 4.7. Metabolic Classification in Pancreatic Cancer

The metabolic environment in PDAC is spatiotemporally heterogeneous, and metabolic heterogeneity is considered an important characteristic of PDAC. The analysis of PDAC cell lines based on their bioenergetic preferences and responses to metabolic inhibitors has demonstrated three metabolic subtypes, namely, “glycolytic”, “lipogenic”, and “slow proliferative” [63]. These subtypes correspond to transcriptional states, as follows: the glycolytic subtype matches the “quasimesenchymal” transcriptional subtype, whereas the lipogenic state aligns with the “classical” subtype identified by Collison et al. [60,122]. A recent analysis of clonal strains isolated from PDAC has shown that two distinct cell populations can coexist within the same tumor [203]. Interestingly, in a tumor sample isolated from a PDAC patient, one cell subtype within the tumor had a constitutively active integrated stress response that was shown to produce asparagine, which was found to support the mitochondrial metabolism in the other cell population within the tumor that showed inhibited respiration [203]. Notably, the depletion of extracellular asparagine by polyethylene-glycolylated L-asparaginase was shown to cause the sensitization of PDAC to mitochondrial targeting by phenformin, which is a mitochondrial complex I inhibitor identified as the most effective metabolic inhibitor from the screening of PDAC human-derived xenografts [204].

Differences in the TME, blood flow, metastatic pathways, and nutrient availability in secondary organs result in the diverse metabolic states of PDAC cells [205]. As mentioned above, glutamine deprivation promotes the EMT by upregulating the EMT regulator Slug, facilitating metastasis to the lungs. A comparison of the epigenetic metabolic states of primary and metastatic PDAC tumors demonstrated the upregulation of the anabolic glucose metabolism and the reliance on oxidative PPP during metastatic progression [206]. This reconstituted metabolic pathway depends on a unique pentose conversion pathway centered on increased phosphogluconate dehydrogenase (PGD) activity [207]. High glycolytic flux maintains an increased PGD metabolism, partially sustained by the transcriptional suppression of thioredoxin-interacting protein [208].

### 4.8. Metabolic Interactions Between Cells

Metabolic symbiotic interactions between stromal cell populations, including immune cells, and tumor cells in the TME promote resistance to chemotherapy and inhibitory effects on antitumor immune surveillance mechanisms via the metabolic activation of PDAC, and not just via nutrient supply.

Fibroblasts, which are the majority of stromal cells in the PDAC TME, selectively release alanine, which is consumed by PDAC cells to promote their metabolism [209]. This exchange of alanine and other nonessential amino acids involves both autophagy and macropinocytosis in PDAC cells [209,210]. Metabolites, such as pyruvate released from cancer-associated fibroblasts (CAFs), help PDAC cells to maintain their redox balance [211].

**Figure 2:**
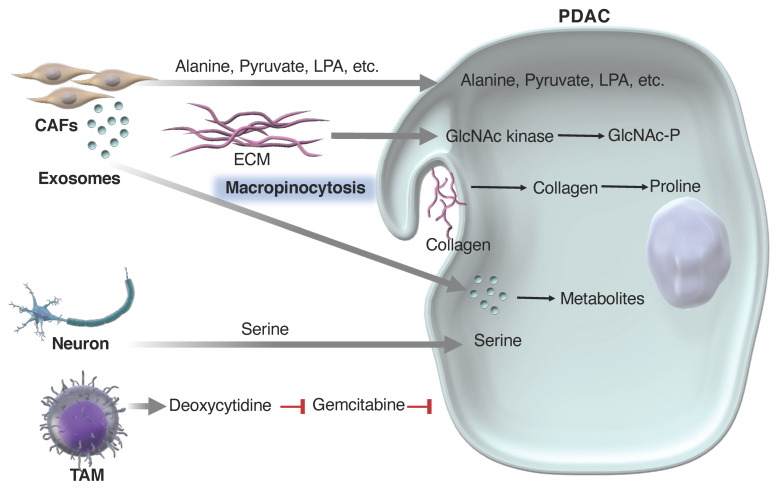
Metabolic interactions within the TME. The metabolic symbiotic interactions between stromal cell populations, including immune cells and tumor cells, in the TME promote resistance to chemotherapy and suppress antitumor immune surveillance mechanisms, not only through nutrient supply but also via the metabolic activation of PDAC. Fibroblasts, which make up the majority of stromal cells, selectively release alanine and pyruvate, and secrete lipids, including LPA, to promote the proliferation and migration of PDAC. Exosomes transport metabolic products and proteins containing amino acids to PDAC cells. PDAC utilizes macropinocytosis to obtain proline from collagen produced by CAFs. PDAC can also acquire GlcNAc through the salvage of N-acetylglucosamine kinase from the ECM. Neurons supply serine and support mRNA translation in PDAC. TAMs and anti-inflammatory macrophages release pyrimidine species, and deoxycytidine derived from the pyrimidine species inhibits the chemotherapeutic effects of gemcitabine. CAF, cancer-associated fibroblast; TAM, tumor-associated macrophage; ECM, extracellular matrix; LPA, lysophosphatidic acid; GlcNAc, N-acetylglucosamine.

Exosomes from CAFs transport metabolites and proteins, including amino acids, to PDAC cells [212,213]. The differential expression of solute carrier proteins (SLCs) facilitates a unidirectional alanine exchange between fibroblast-SLC1A4 and PDAC-SLC38A2 [214]. Activated CAFs from stellate cells secrete several lipid species, including lysophosphatidylcholine, which is hydrolyzed by autotaxin to produce lysophosphatidic acid (LPA), promoting PDAC proliferation and migration [215]. PDAC also programs CAFs to produce branched-chain α-keto acid precursors, which are used in the PDAC metabolism [216]. In addition to the direct metabolic exchange, components of the ECM from fibroblasts and PDAC cells are used in the PDAC metabolism. PDAC uses macropinocytosis to obtain proline from CAF-produced collagen [217]. PDAC can also acquire N-acetylglucosamine (GlcNAc) by N-acetylglucosamine kinase salvage from the ECM [218] (Figure 2).

Tumor-associated macrophages (TAMs) and anti-inflammatory macrophages release pyrimidine species, which are preferentially consumed by PDAC cells [219]. Deoxycytidine from these pyrimidines inhibits the effects of gemcitabine chemotherapy, with PDAC tumors in mice becoming sensitized to gemcitabine when myeloid cells are depleted. Deoxycytidine from TAMs and CAFs inhibits the effects of gemcitabine through molecular competition at deoxycytidine kinases [219,220] (Figure 2).

Perineural invasion is common in PDAC tumors and is associated with an unfavorable disease prognosis [221]. Recently, it has been demonstrated that sensory nerves release serine into the TME of PDAC, supporting the growth of serine-dependent tumors [222]. PDAC cells that lack enzymes for serine biosynthesis rely on external serine (exSer) for their proliferation. Neurons supply serine to PDAC cells in nutrient-deprived conditions, thereby rescuing their growth. Serine deprivation reduces mRNA translation efficiency, particularly for specific serine codons (TCC and TCT), causing ribosome stalling and disrupting protein synthesis. Interestingly, nerve growth factor (NGF) was less affected by serine codon bias and was secreted in higher amounts by exSer-dependent PDAC cells under serine deprivation than in normal conditions. In vivo, a serine–glycine-free diet was found to increase tumor innervation, whereas NGF receptor inhibition reduced tumor growth and innervation [222]. In patients, the low expression of the serine biosynthesis enzyme phosphoglycerate dehydrogenase correlated with higher tumor innervation and a less favorable prognosis, indicating neuronal metabolic support as a potential therapeutic target for PDAC [222] (Figure 2).

These studies show that targeting the cancer cell metabolism as a therapy involves more than identifying their vulnerabilities. The metabolic state of PDAC cells is heterogeneous within tumors. Understanding the symbiotic nutrient support mechanisms in the pancreatic TME has identified therapeutic opportunities, which will expand as our knowledge of the PDAC metabolic crosstalk network grows.

## 5. Immunosuppressive TME in PDAC

**Figure 3:**
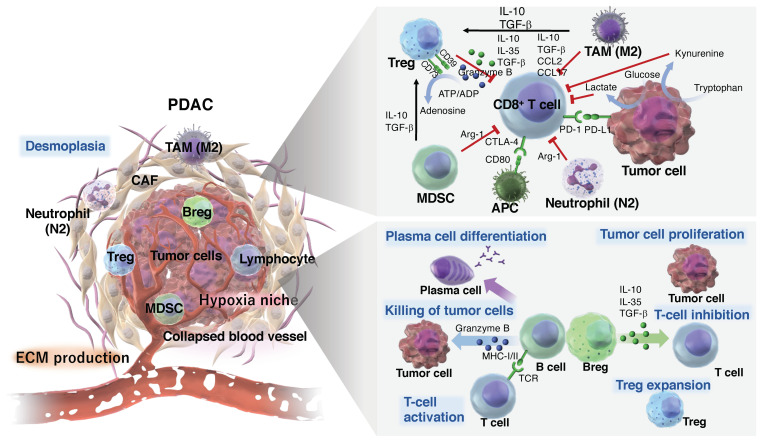
Immunosuppressive TME in pancreatic cancer. In PDAC, the TME demonstrates substantial desmoplasia, leading to the disruption of antitumor mechanisms and the development of immunosuppressive properties, which result in treatment resistance. CAFs produce numerous ECM components, such as collagen, making the tumor stroma very rigid, which crushes blood vessels under this pressure and induces a hypoxic state. The TME contains a large number of infiltrating myeloid cells, including monocytes, macrophages, dendritic cells, and granulocytes. Tregs, MDSCs, neutrophils (N2), and TAMs (M2) inhibit T-cell responses. Neutrophils differentiate into immune-stimulating (N1-like) or immune-suppressive (N2-like) subtypes depending on their activation state, each with different functions. N1-like neutrophils produce chemokines that recruit T-cells, whereas N2-like neutrophils increase the production of ARG-1, which suppresses T-cell function. TAMs demonstrate both tumor-promoting roles (M2) and antitumor roles (M1) depending on their macrophage polarization phenotype. APCs and tumor cells inhibit T-cell responses through immune checkpoint molecules. Tumor cells also suppress T-cell responses via lactate and kynurenine. TIL-Bs consist of various subtypes, including effector B-cells and Bregs, which can demonstrate both antitumor and tumor-promoting activities. Bregs exert inhibitory effects on CD4^+^ and CD8^+^ T-cells, dendritic cells, and monocytes, whereas they support the activity of Treg cells. B-cells exert antitumor activity by differentiating into antibody-secreting plasma cells, presenting antigens to T-cells, and secreting granzyme B. CAF, cancer-associated fibroblast; TAM, tumor-associated macrophage; Breg, regulatory B-cell: Treg, regulatory T-cell; MDSC, myeloid-derived suppressor cell; APC, antigen-presenting cell.

The TME is an ecosystem created by cancer cells, and is composed of factors contributed by both the tumor and the host. Various factors present in the TME ensure tumor development, progression, and expansion by hindering the supply of nutrients and oxygen, preventing immune surveillance, and obstructing the efficient delivery of drugs [62,223]. The dynamic interaction between these cellular and extracellular components of cancer cells and the TME is essential for tumor cell heterogeneity, clonal evolution, and the enhancement of multidrug resistance [224]. Therefore, the improvement in tumor immunotherapy techniques is closely associated with an improved understanding of the TME. In PDAC, the TME is highly fibrotic and known to induce treatment resistance owing to its immunosuppressive nature, which disrupts antitumor mechanisms (Figure 3). This section discusses the immune composition of that environment.

### 5.1. Innate Immune Cells

#### 5.1.1. Neutrophils

Neutrophils are a crucial type of innate immune cell that infiltrates the PDAC TME. The percentage of neutrophils infiltrating pancreatic tumors is known to be higher than both healthy pancreatic tissue and pancreatic tissue with chronic pancreatitis [225]. In humans, neutrophils are identified as being positive for CD66b, CD177, and CD15 [225,226], whereas, in mouse models, they are positive for Gr1 (granulocyte receptor 1) and Ly6G (lymphocyte antigen 6 complex, locus G) [227]. Several studies have demonstrated that the C-X-C motif chemokine ligands CXCL1–3, CXCL5–6, and CXCL8 interact with the C-X-C motif chemokine receptors CXCR1 and CXCR2 expressed on neutrophils, which contributes to their recruitment to the TME [227,228]. Additionally, PDAC tumor cells are known to secrete chemokines, including CXCL1, granulocyte colony-stimulating factor (G-CSF), granulocyte/macrophage colony-stimulating factor, and CXCL16, which promote neutrophil migration [229]. TGF-β [230] and IL-17 [231] have also been demonstrated to stimulate the mobilization of neutrophils.

In general, in the case of PDAC, neutrophils in the pancreas during the PanIN stage demonstrate high migratory ability without immunosuppressive properties. However, in the later stages of the disease, neutrophils become immunosuppressive by inhibiting T-cell proliferation, and their migratory ability decreases [232]. This indicates the heterogeneous nature of neutrophils within the TME. Immature neutrophils more readily infiltrate the PDAC TME than the healthy pancreas. Additionally, tumor burden is known to positively correlate with the quantity of immature neutrophils present in the blood and pancreas [233]. Neutrophils have also been proposed to differentiate into immunostimulatory (N1-like) or immunosuppressive (N2-like) subtypes, depending on their activation state, each with distinct functions. In terms of their function, N1-like neutrophils secrete chemokines that attract T-cells, whereas N2-like neutrophils enhance the production of arginase 1 (ARG-1), which inhibits T-cell activity [234] (Figure 3). Whereas TGF-β [234] and G-CSF [235] are known to promote an immunosuppressive phenotype in neutrophils, other factors affecting neutrophil polarization are still being investigated. Further research is required to elucidate how neutrophils are polarized and their roles within the TME.

#### 5.1.2. Myeloid Cells

The TME is populated with a diverse array of myeloid cells, such as monocytes, macrophages, dendritic cells (DCs), and granulocytes. These cells demonstrate a range of functions within the TME, from immunosuppressive to immunostimulatory roles. Using advanced technologies to profile immune cells within the TME of patients, substantial differences were shown in the immune cell infiltration and composition of the TME, particularly regarding myeloid cells, even among patients with the same tumor type [62,236,237,238]. Myeloid cells within the TME are characterized by their diversity, including both mononuclear cells (monocytes, macrophages, and DCs) and polymorphonuclear granulocytes [62,239,240,241]. In healthy tissues, these myeloid cells contribute to tissue repair and act as the initial defense against threats, such as pathogens and viruses. In addition, these myeloid cells are heterogeneous in both their form and function, which likely facilitates a range of responses to the various challenges encountered in both normal and pathological physiologies. Within the TME, these cells can either inhibit or enhance antitumor immunity, and their crucial functions include phagocytosis and antigen presentation to T-cells [240,242,243].

The most abundant myeloid population in tumors is generally known to be TAMs [243,244]. TAMs are frequently categorized into two distinct states, i.e., classical activation (“M1”) or alternative activation (“M2”), based on in vitro studies in which macrophage differentiation was modified by individual chemokines, such as interferon γ (IFN-γ) and IL-4 [245]. M1 macrophages are induced in the TME by cytokines, such as IFNγ and TNFα, where they secrete inflammatory cytokines, chemokines, and effector molecules, enhancing the antitumor activity. On the other hand, M2 macrophages produce cytokines and chemokines, such as IL-10, TGF-β, CCL2, and CCL17, inhibiting the activity of CD8^+^ cytotoxic T-cells and NK-cells while promoting the migration of regulatory T-cells (Tregs) into the TME (Figure 3). The “M2” signature is sometimes associated with an unfavorable disease prognosis, but tends to be associated with anti-inflammatory responses, wound-healing processes, and tissue remodeling [245].

To assess the effectiveness of targeting macrophage populations within the TME, it is crucial to gain a deeper understanding of macrophage origins and their diversity. Recent research utilizing scRNA-seq has demonstrated that macrophage heterogeneity and plasticity is more complex than initially thought, which could affect tumor progression and responses to immunotherapy [236,237,238].

### 5.2. Cells of the Acquired Immune System

T-cell dysfunction within the TME is a hallmark of cancer [246]. To overcome this dysfunction, immunotherapies, such as the adoptive transfer of genetically modified T-cells and immune checkpoint inhibitors, have emerged as new treatments for advanced cancers. However, some patients show resistance or tolerance to these treatments, and the underlying mechanisms of tumor immune resistance are not fully understood at present.

#### 5.2.1. T-Cells

T-lymphocytes are the most effective immune defense tools against cancer of the host. They specifically identify tumor-associated antigens and attack tumor cells [247,248,249,250], exerting direct antitumor effects. Additionally, they enhance immune responses through the release of lymphokines.

To conduct thorough immune surveillance and initiate strong immune responses, circulating and tissue-resident T-cells have various immunological regulatory mechanisms for maintaining immune homeostasis. These regulatory mechanisms involve the T-cell expression of inhibitory proteins, such as PD-1, T-cell immunoglobulin (Ig) mucin domain-containing protein-3 (TIM3), lymphocyte activation gene-3 (LAG3), T-cell immunoreceptor with Ig and immunoreceptor tyrosine-based inhibitory motif domains (TIGIT), sialic acid-binding Ig-like lectins (Siglecs), and CTLA-4, which are commonly referred to as “immune checkpoints” [246,251]. These inhibitory proteins and suppressive cells primarily function to maintain self-tolerance and mitigate the risk of autoimmunity, yet they also have the ability to obstruct the host’s antitumor response within the TME [246]. However, beyond immune checkpoints, there are also obstacles that create a complex TME that preferentially restricts the function of TILs [252]. These include the increase in immunosuppressive metabolic products, hypoxia, and lactate, and the dysregulation of bioenergetic signals. For example, when abnormal vasculature formation and reduced blood flow restrict oxygen delivery, a hypoxic environment is created. Hypoxia attracts Tregs, which are a subset of immunosuppressive cells in the TME, and impedes tumor-specific T-cell responses [252]. The mechanisms by which Tregs suppress effector T-cell responses include the direct destruction of effector T-cells through the action of granzymes and perforin, the secretion of suppressive cytokines such as IL-10 and TGF-β, the suppression of CD8^+^ effector T-cells via membrane-bound TGF-β, competition for access to antigen-presenting DCs, and the inhibition of the cellular metabolism through the promotion of adenosine production [253] (Figure 3). As a result, the function of effector T-cells is suppressed, promoting tumor growth. Hypoxia further increases glucose uptake and glycolysis in cancer cells, worsening the glucose shortage in the TME [254].

PDAC tumors are often considered immunologically ‘cold’ tumors. In the TME of PDAC, there is an abundance of Tregs, TAMs, and MDSCs that are involved in suppressing antitumor immune responses [255]. In contrast, antitumor CD4^+^ and CD8^+^ T-cells, NK-cells, and DCs are relatively scarce [256,257]. Other T-cell populations within the TME, such as T helper 17 (Th17), Th22, and γδT cells, have all been noted to play tumor-promoting roles [258,259,260].

Conventional cytotoxic CD8^+^ T-cells are effective in orchestrating antitumor immune responses and are able to directly destroy cancer cells by releasing granules with enzymes, such as granzymes and perforin. Tumor infiltration by CD8^+^ T-cells is associated with a more favorable prognosis in various cancer types, including PDAC [261,262]. Although considerable attention has been given to CD8^+^ T-cells, the importance of CD4^+^ T-cells in tumor management and responses to immunotherapeutic approaches is also becoming increasingly evident [263]. CD4^+^ helper T-cells authorize antigen-presenting cells (APCs) to effectively prime CD8^+^ T-cells and activate NK-cells, myeloid cells, and other cell types through secreted factors. Recent findings suggest that a specific subset of CD4^+^ T-cells has cytotoxic properties and can directly eliminate cancer cells [263].

Conventional subsets of CD4^+^ T-cells, including Th1, Th2, Th17, Th9, follicular helper T, and Treg cells, are present within tumors, and imbalances in these subsets are implicated in various pathological conditions [264,265,266,267]. CD4^+^ T-cells with the Th1 phenotype play a crucial role in antitumor responses, and the number of these cells is associated with favorable outcomes. In addition to the recruited T-cell population, tissue-resident memory T-cells (TRMs), which are tissue-specific constituents of the TME, also exist. TRMs, which can be either CD4^+^ or CD8^+^ T-lymphocytes, stay in tissues for a long time after the primary T-cell response, and the proportion of T-cell infiltration is associated with a favorable prognosis [268]. Recent research on TILs in PDAC has identified substantial populations of CD8^+^ TRM, Treg, and Th17 cells with an exhausted phenotype (high PD1 and high TIGIT expression), consistent with an immunosuppressed microenvironment [269]. Furthermore, infiltration by both CD4^+^ and CD8^+^ T-cells has been shown to be linked to longer overall survival and disease-free survival in PDAC patients. However, the TME of PDAC is characterized by the limited infiltration of CD4^+^ lymphocytes and CD8^+^ lymphocytes [270,271].

It is not yet fully understood when and how tumor-specific T-cells differentiate into a less responsive state during tumor development in patients, and how this state changes over time. Recent technological advancements have provided substantial insights into the heterogeneity of CD8^+^ TILs, demonstrating the existence of T-cell subsets with distinct functional states that respond differently to therapeutic reprogramming [246]. Identifying tumor-reactive T-cell populations and their functional states in patients, and comprehending how these T-cells perform antitumor effector functions, are crucial challenges for understanding and predicting the efficacy of immunotherapy strategies.

#### 5.2.2. B-Cells

The involvement of B-cells in either promoting or suppressing tumor development through immunological mechanisms has become increasingly recognized [272,273,274].

Tumor-infiltrating B-lymphocytes (TIL-Bs) are a major component of TILs in cancer, consisting of various phenotypes, including subsets of effector B-cells and regulatory B-cells (Bregs), and can show both tumor-promoting and antitumor activities [275,276,277]. The dual role of B-cells has been demonstrated to be affected by several factors, such as hypoxia, other immune cells (e.g., Tregs and MDSCs), cytokines, and metabolic products produced by tumor cells [278]. Generally, TIL-Bs, together with T-cells, NK-cells, and myeloid cells, are present in the TME and show active antigen recognition and diverse effector functions that contribute to the suppression of tumor growth [279,280,281]. On the other hand, Bregs are considered to be tumor-promoting immune cells, and have been demonstrated to support tumor growth and metastasis [282,283,284]. Bregs have been shown to suppress inflammatory cells, such as monocytes [285], DCs [286], various CD4^+^ T-cell subsets [287,288,289], and CD8^+^ T-cells [290,291], through the secretion of cytokines, such as IL-10, IL-35, and TGF-β, as well as by releasing metabolic products such as gamma-aminobutyric acid (Figure 3). Furthermore, Bregs can directly inhibit CD4^+^ T-cells and CD8^+^ T-cells through the PD-1/programmed death ligand 1 (PD-L1) [292,293] and CTLA-4 pathways [294].

Interestingly, PDAC patients show an increase in Bregs compared with healthy individuals, and the level of Bregs in peripheral blood has been reported to correlate with advanced tumor stage and reduced survival outcomes [295]. In animal models, it has been proposed that Bregs and PDAC cells interact in a mutually activating manner, contributing to tumor progression. Specifically, pancreatic cancer cells promote the proliferation of Bregs through the secretion of IL-18, and, in turn, Bregs aid in immune evasion and tumor growth by expressing PD-L1 and IL-35 [295,296,297]. Bregs in PDAC patients also show increased levels of PD-L1 and interact with CD8^+^ T-cells expressing PD-1 in vitro, leading to a reduction in their proliferation and IFN-γ secretion [292,296]. The simultaneous inhibition of the IL-18 and PD-L1/PD-1 pathways using synthetic inhibitors leads to reduced PDAC growth and metastasis in orthotopic animal models, highlighting the in vivo significance of Breg–cancer cell crosstalk in the progression of pancreatic cancer [295].

Moreover, the activation of Bruton’s tyrosine kinase (BTK) in TIL-Bs has been found to drive the reprogramming of tumor-infiltrating macrophages to the M2 phenotype, impairing CD8^+^ T-cell-mediated antitumor responses, and contributing to the progression of PDAC [298,299]. Specifically, BTK shows higher activation in CD20^+^ B-lymphocytes within PDAC than peripheral leukocytes, playing a crucial role in Breg cell differentiation and the mobilization of myeloid cells to the tumor site [300,301]. Therefore, B-cells are becoming increasingly attractive therapeutic targets in the intricate pathophysiology of pancreatic cancer.

### 5.3. Nonimmune Cells

Fibroblasts are present in all organs of the human body, and are essential cells for maintaining organ shape and structure. It is known that, when cancer occurs, various factors produced by cancer cells cause an increase in fibroblasts. These fibroblasts are referred to as CAFs and constitute a major component of the TME. CAFs also produce numerous growth factors, which promote the proliferation and invasion of cancer cells while suppressing the antitumor immune response. Many studies have shown that CAFs are involved in cancer malignancy and treatment resistance [302,303].

In invasive cancers such as PDAC, it has been reported that the tumor is composed of a high-density fibrous stroma that accounts for 90% of the tumor volume [304,305]. The presence of a high-density fibrotic barrier, known as desmoplasia, is an important factor affecting the development, progression, metastasis, and treatment resistance of PDAC [303,306].

For example, CAFs produce a large amount of ECM components, such as collagen, which makes the cancer stroma very stiff. As a result, blood vessels become collapsed (compressed) by this pressure, leading to the hindrance of the efficient penetration of anticancer agents. The compression of blood vessels is known to induce a hypoxic state in cancerous tissue, which further increases the malignancy of cancer cells. Furthermore, the exacerbation of inflammation, owing to the influx of inflammatory cells and cytokines, forms an inherently immunosuppressive TME with a low immunogenicity [307].

Collagen is the most prominent protein component of the ECM of PDAC, constituting more than 90% of the ECM matrix throughout all stages of the disease. These ECM proteins are overexpressed in PDAC [308,309]. Furthermore, the increased collagen content in PDAC has been shown to correlate with the decreased survival rates of PDAC patients [307]. Increased collagen thickness and tissue tension have also been shown to be associated with the prognosis of PDAC [310].

Notably, focal adhesion kinase (FAK), which is hyperactivated in pancreatic cancer cells, plays a crucial role in driving fibrosis and immunosuppression within the TME [311]. Additionally, combination therapy of FAK inhibition and immune checkpoint blockade has shown to cause tumor regression in mouse models of aggressive PDAC, suggesting the potential clinical application of this approach. Moreover, recent studies have shown that stromal reprogramming via FAK inhibition can counteract radiation resistance and enhance the effectiveness of immunotherapy in tumors [312].

However, the desmoplastic collagen in PDAC does not always promote tumor progression; it can also act as a barrier to carcinogenesis. For instance, it has been shown that reducing desmoplasia in the stroma of PDAC tumors by inhibiting the Sonic Hedgehog pathway, which is crucial for paracrine signaling between epithelial cells and fibroblasts, promotes tumor growth and metastasis [313,314]. Furthermore, the genetic depletion of alpha smooth muscle actin (αSMA)-positive CAFs in mouse PDAC models was found to lead to the progression of PDAC, and the reduction in myofibroblasts in human PDAC has also been shown to correlate with decreased patient survival rates, suggesting that specific subsets of CAFs may have functions that inhibit cancer progression [304,313,315]. Overall, these findings clearly indicate that targeting CAFs should be approached with caution.

Importantly, recent observations have indicated that the functions of the various CAF subtypes are not necessarily identical [303,304,313,314,315,316,317,318]. The heterogeneity of CAFs in PDAC tumors is a rapidly advancing area of research, particularly with the development of single-cell technologies. Current perspectives indicate the existence of heterogeneous CAF populations, comprising both cancer-promoting and cancer-inhibiting CAFs [319]. Future developments are anticipated in the biological research of CAFs, including their origins, functions, and clinical relevance.

## 6. Microbiota

The intricate and evolving buildup of microorganisms within the body, also referred to as the microbiome, has recently attracted scientific attention for its role in the development of various diseases, including pancreatic cancer.

According to Whipps et al. in 1988, the microbiome refers to a ‘characteristic microbial community occupying a reasonably well-defined habitat with distinct physiochemical properties’ [320,321]. It is well known that an extremely large number of microorganisms reside in the colon [322]. In healthy individuals, the microbiome performs numerous physiological functions that contribute to the organism’s homeostasis [323,324]. The discovery that tumors also harbor their own unique microbiome, which varies by tumor type and differs from that of healthy tissue, was groundbreaking [325,326,327]. It is now widely accepted that approximately 20% of all cancers are associated with specific viral or microbial infections [328]. Whereas the heterogeneity of the microbiome might affect tumor prognosis, it is becoming increasingly clear that the presence of certain taxa within the microbiome of a tumor is linked to either favorable or unfavorable outcomes [329,330]. In recent years, microorganisms have emerged as key factors linking immune responses and cancer [328]. Additionally, it is recognized that the microbiome plays crucial roles in interactions with the immune system and cancer therapies [329,331,332,333,334]. In 2022, Hanahan released an updated comprehensive review on cancer research, adding the microbiome as a new hallmark of cancer [115].

To date, there are increasing lines of evidence indicating that certain bacteria are found in PDAC, that the microbiome of pancreatic tumors may share common attributes with the gut microbiome, and that the microbiome in PDAC is distinctly different from that in normal pancreatic tissue [325,326,327,329,330,335,336,337,338]. In addition, research using a PDAC mouse model demonstrated that microbiome depletion enabled the modulation of the immune cell composition, including a decrease in bone marrow-derived suppressor cells and an increase in the activation of Th1-type CD4^+^ and cytotoxic CD8^+^ T-cells, resulting in a substantial decrease in tumor burden in the mice [325]. Another group linked the abundance of microbiota in PDAC patients to immunosuppression, including decreases in T-cells and M2 macrophages, the activation of cancer-associated pathways, and the reduction in tumor-suppressive pathways. They also identified 13 microbiota that are associated with abnormal regulatory gene signatures, including those associated with carcinogenic methylation, cancer progression, and immune regulation [339].

In recent years, associations between the microbiome and the tumor immune system are becoming clearer and may provide new insights for therapeutic strategies. Nevertheless, the precise composition of the tumor microbiota remains unclear. As the function of the microbiome in PDAC becomes clearer through refined approaches, the information obtained from tumor genomes and the composition of the gut microbiota might enable the assessment of the cancer disease state and disease prognosis.

## 7. ARF6, KRAS Mutations, Metabolic Rewiring, Mesenchymal Plasticity, and Immune Evasion

**Figure 4:**
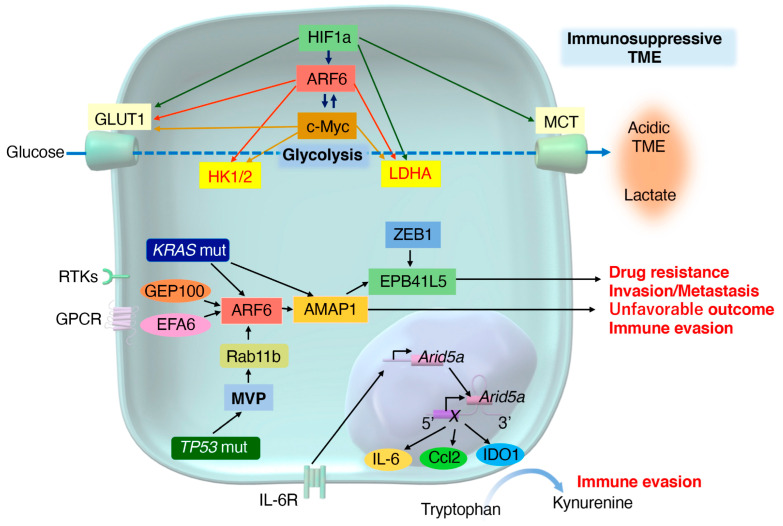
Involvement of the ARF6-AMAP1 pathway and Arid5a in cancer progression and immune evasion. ARF6 is activated by GEP100 or EFA6 through activated RTKs or GPCRs, and transmits signals via its interaction with AMAP1, an effector of ARF6, contributing to cancer malignancies, such as invasion, metastasis, drug resistance, immune evasion, and unfavorable outcomes. In PDAC, mutations in *KRAS* and *TP53* promote cancer malignancy through the ARF6-AMAP1 pathway. *KRAS* mutations (*KRAS* mut) drive the upregulation of ARF6 and AMAP1 during the translation process. The upregulation of the MVP owing to *TP53* mutations (*TP53* mut) is involved in the activation of ARF6 through the geranylgeranylation modification of Rab11b. EPB41L5, induced by EMT, interacts with AMAP1 and is involved in the acquisition of mesenchymal traits. Additionally, ARF6 plays a role in regulating the expression of GLUT1, HK2, LDHA, and c-Myc. *ARF6* mRNA levels significantly increase with the activation of HIF-1α and are also associated with the EMT. c-Myc enhances the expression of several key glycolytic genes, including *GLUT1*, *HK2*, and *LDHA*, and is also associated with the transcriptional activation of ARF6. The RNA-binding protein Arid5a stabilizes *IL-6*, *Ccl2*, *IDO1*, and other mRNAs, playing diverse roles in immune regulation and tumor progression. IL-6 increases the level of Arid5a, enhancing the stability of its mRNA and forming a positive feedback loop. The stabilization of *Ido1* mRNA by Arid5a reduces the concentration of tryptophan in the tumor, leading to the production of kynurenine, which promotes the differentiation and activation of Tregs, resulting in an immunosuppressive environment. The stabilization of *Ccl2* mRNA by Arid5a induces immunosuppressive cells in the tumor, contributing to an immunosuppressive environment. Thus, the ARF6-AMAP1 pathway and Arid5a play crucial roles in cancer malignancy, highlighting their significance in the complex process of tumor progression. GPCR, G-protein-coupled receptor; HK1/2, hexokinase1/2; IDO, indoleamine 2,3-dioxygenase; LDHA, lactate dehydrogenase A; MCT, monocarboxylate transporter; MVP, mevalonate pathway; RTK, receptor tyrosine kinase.

ARF6 is a small GTPase with various physiological functions. ARF6 is expressed in a broad range of cells and tissues, and is predominantly found at the plasma membrane and recycling endosome compartments. It plays a key role in regulating intracellular dynamics associated with membrane recycling (both endocytosis and recycling to the plasma membrane), and actin cytoskeleton reorganization at the cell periphery [340,341]. Recently, it has been reported that silencing ARF6 in *KRAS*-mutant PDAC cells reduces the Warburg effect linked to aerobic glycolysis [342]. The oncogene c-Myc functions as a transcription factor that controls aerobic glycolysis by enhancing the expression of several key glycolytic genes, including *GLUT1*, *HK2*, and *LDHA* [85,343], and is linked to the transcriptional activation of ARF6. ARF6 has also been demonstrated to play a role in regulating the expression of *GLUT1*, *HK2*, *LDHA*, and *c-Myc* [342] (Figure 4). Therefore, ARF6 may be involved in the regulation of aerobic glycolysis by modulating c-Myc in PDAC. Interestingly, it has been shown that *ARF6* mRNA levels significantly increase with the activation of HIF-1α [344], and ARF6 activity is markedly enhanced under hypoxic conditions [345]. As mentioned above, HIF-1α has been shown to be involved in the gene expression of a group of enzymes involved in glycolysis and in the EMT induced in tumors [346], suggesting that HIF-1α and ARF6 may cooperatively act to reprogram the PDAC metabolism and to acquire invasive and metastatic traits.

We have demonstrated that ARF6-centered signaling pathways play an executory role in the invasiveness and metastasis of malignant cancers that have acquired mesenchymal traits and immune evasiveness [347,348,349]. For example, the ARF6-specific guanine nucleotide exchange factor (GEF) GEP100 (also known as IQ motif and Sec7 domain ArfGEF 1) is attracted to ligand-activated receptor tyrosine kinases (RTKs), such as EGFR, erb-b2 receptor tyrosine kinase 2, platelet-derived growth factor receptor beta β, and vascular endothelial growth factor receptor 2, leading to the activation of ARF6 through the conversion from ARF6-GDP to ARF6-GTP [350,351,352,353]. Another GEF, exchange factor for Arf6 (EFA6), which interacts with the GTP-bound G-protein subunit alpha 12 released from G-protein-coupled receptors mediated by lysophosphatidic acid, also activates ARF6 in clear cell renal cell carcinoma [354] (Figure 4).

Activated ARF6 transmits signals through its effector, AMAP1 (also known as ArfGAP with the SH3 domain, ankyrin repeat, and PH domain 1). AMAP1 contains several protein interaction motifs, including Src homology 3 domains and proline-rich domains, which are involved in promoting actin reorganization and integrin recycling associated with tumor cell motility [355,356]. AMAP1 also interacts with erythrocyte membrane protein band 4.1-like 5 (EPB41L5), which is a mesenchymal-specific protein induced by the EMT, promoting the internalization of E-cadherin and the dynamics of focal adhesion, thus enhancing cell motility [357]. The *EPB41L5* gene has been shown to be transcriptionally activated by the EMT master regulator zinc finger E-box-binding homeobox 1 (ZEB1) [358]. Interestingly, ZEB1 is highly expressed in the quasimesenchymal subtype of PDAC, and has been reported to be involved in the malignancy of this subtype [359]. Consistent with this, we demonstrated that the expression levels of ARF6, AMAP1, and EPB41L5 are high in quasimesenchymal subtype PDACs [358]. Furthermore, in the Cancer Genome Atlas RNASeq dataset of human primary breast cancer (n = 970), increased *ZEB1* mRNA levels were found to be statistically associated with increased *EPB41L5* mRNA levels [358]. These findings suggest that the ARF6-centered signaling pathway mediated by the ZEB1-induced EMT is associated with mesenchymal plasticity in PDAC.

Small G-proteins, such as KRAS and ARF6, require lipid modifications to localize to intracellular membrane components for their functions. These lipid modifications, namely, geranylgeranylation and farnesylation, utilize intermediate metabolites of the MVP. Notably, mutant p53 has been shown to promote tumor formation through activation of the MVP [360]. We found that the upregulation of the MVP owing to *TP53* mutations enhances the activation of ARF6 [361]. Mechanistically, ARF6 is transported to the plasma membrane through the lipid modification of Rab11b mediated by the MVP enzyme geranylgeranyltransferase II (GGT-II), and becomes activated through receptor tyrosine kinases in response to external ligands. Inhibiting GGT-II not only suppressed tumor invasion and metastasis, but also diminished resistance to chemotherapeutic drugs in tumor cells that overexpress components of the ARF6 pathway. Furthermore, statins, which are MVP inhibitors, inhibited ARF6 activation and invasiveness in PDAC cells and decreased the resistance of tumor cells to chemotherapeutic drugs [361]. Thus, the ARF6-based mesenchymal pathway appears to enhance tumor malignancy in PDAC in conjunction with the TP53 mutation–MVP activation axis (Figure 4).

We discovered that mutant KRAS is involved in upregulating the expression of ARF6 and AMAP1 by translation processes through different mechanisms. The 5′-untranslated regions (UTRs) of *ARF6* and *AMAP1* mRNAs have a high G/C content, and ARF6 has a G-quadruplex structure in its 5′-UTR, indicating the necessity of RNA helicase eukaryotic translation initiation factor 4A (eIF4A) activity to unwind this structure for translation [362,363]. AMAP1 has an oligopyrimidine-like sequence at its 5′-end, indicating its regulation by mTOR and eIF4E [364,365]. Indeed, we discovered that ARF6 expression is markedly sensitive to the eIF4A inhibitor silvestrol. Mechanistically, we showed that mutant KRAS suppresses the expression of programmed cell death 4 (PDCD4), which directly binds to and inhibits eIF4A function through the induction of transcription factors such as TEA domain transcription factor 3 (TEAD3) and ETS variant transcription factor 4 (ETV4). On the other hand, AMAP1 translation is substantially reduced by the silencing of mTOR and the regulatory-associated protein of mTOR complex 1 (RPTOR), and mTOR inhibitors, such as rapamycin and Torin 1, decrease AMAP1 levels, whereas ARF6 levels remain unaffected [352].

In animal experiments using KPC cells derived from KPC (LSL-Kras[G12D/+]; LSL-Trp53[R172H/+]; Pdx-1-Cre; a genetically engineered mouse model of PDAC) mice harboring KRAS and TP53 mutations, which was established as a model of human malignant PDAC, we observed that suppressing the expression of molecules constituting the ARF6-AMAP1 pathway had no effect on tumor formation in immunodeficient mice, but significantly suppressed tumor formation in syngeneic immunocompetent mice [352]. This finding suggests that there is a strong connection between the ARF6-AMAP1 pathway and immune evasion in PDAC cells. We demonstrated that one molecular mechanism is the activation of the ARF6-AMAP1 pathway by PDGFβ, which controls the intracellular dynamics of PD-L1 in PDAC, and particularly promotes recycling to the plasma membrane [352]. Furthermore, using clinical samples from PDAC patients, the high expression of the AMAP1 protein statistically correlated not only with the progression of fibrosis, but also with the high expression of PD-L1 [366]. Consistently, the silencing of AMAP1 in KPC cells was found to decrease PD-L1 expression and fibrosis in tumor tissues [366]. Therefore, the upregulation of the intracellular dynamics and gene expression of PD-L1, as well as the promotion of fibrosis, may be part of the underlying mechanism by which the ARF6-AMAP1 pathway induces tumor immune evasion.

Furthermore, to investigate the potential application of targeting the ARF6 pathway in immunotherapy, we conducted experiments, in collaboration with Ono Pharmaceutical, using a tumor formation model with KPC cells transplanted into syngeneic mice so to evaluate the anticancer effect of ICIs. The synergistic effect of anti-PD-1 antibodies and AMAP1 silencing showed stronger tumor suppression, exceeding the tumor suppression observed using the anti-PD-1 antibody alone [367]. Subsequently, we investigated the effects of silvestrol, which was identified as an ARF6 translation inhibitor, and found that silvestrol alone did not demonstrate antitumor activity against PDAC cells but showed a strong synergistic effect when combined with anti-PD-1 antibodies [367]. These findings strongly suggest that targeting the ARF6-AMAP1 pathway partially alleviates the immune evasion of KPC tumor cells, and the combination of the inhibition of this pathway with anti-PD-1 antibody therapy enhances the therapeutic effect.

## 8. Arid5a as a Master Regulator of Immune Modulation and Tumor Plasticity

Arid5a is an RNA-binding protein that directly attaches to the 3′-UTR of the inflammatory cytokine *Il6*, which leads to the stabilization of *Il6* mRNA [368] (Figure 4). Arid5a plays a crucial role in both innate and adaptive immune responses, with its expression in macrophages and embryonic fibroblasts being induced by IL-1, IL-6, and lipopolysaccharide (LPS) [368,369,370]. Increased Arid5a levels in the CD4^+^ T-cells of untreated rheumatoid arthritis (RA) patients are reduced by the anti-IL-6 receptor antibody tocilizumab, implicating the contribution of the IL-6-ARID5A axis in RA pathogenesis [371]. Arid5a affects various immune-associated conditions, including reducing IL-6 production during LPS-induced endotoxemia. In an experimental autoimmune encephalomyelitis (EAE) model, the lack of Arid5a decreased IL-6 serum levels and Th17 cell differentiation, limiting EAE progression [368]. Arid5a deficiency was also shown to stabilize other immune-regulating mRNAs, such as *signal transducer and activator of transcription 3* (*Stat3*), *T-box transcription factor 21*, *Ox40* (also known as *TNFRSF4*), and *Il17* [372,373,374,375]. IL-6 enhances the stability of its own mRNA by increasing Arid5a levels, forming a positive feedback loop [376] (Figure 4). Mice lacking Arid5a show weakened responses to LPS, with the reduced expression of *Il6* and *Ifnγ*, and the resistance to lethal endotoxic shock, indicating the role of Arid5a in enhancing Th1 and Th17 cell functions in inflammation and autoimmune diseases [368,373].

Arid5a mRNA and protein levels are markedly increased in mesenchymal subtypes of PDAC and colorectal cancer (CRC) [377]. In PDAC models, Arid5a deletion reduces EMT-associated transcription factors and markers while increasing the level of the epithelial marker E-cadherin. Pathway analysis showed diminished EMT and metastasis-associated signaling pathways in the absence of Arid5a [377]. Arid5a deficiency in KPC cells also leads to the downregulation of IL-6, STAT3, and Janus kinase (JAK)/STAT pathways. Arid5a expression increases in vitro models of EMT triggered by IL-6 and TGF-β, promoting mesenchymal traits [377]. Consistently, the IL-6-Arid5a axis enhances breast cancer cell invasion and metastasis by upregulating the long noncoding RNA AU021063, which promotes cell invasion and metastasis through the stabilization of tribbles homolog 3 mRNA and the activation of the Mek/Erk signaling pathway [378].

Arid5a also facilitates immune evasion in PDAC and CRC by promoting the mobilization of immunosuppressive granulocytic myeloid-derived suppressor cells (gMDSCs) and Tregs into the TME, while reducing the attraction and activation of antitumor effector T-cells [377]. Arid5a functions as a dual regulator, creating immunosuppressive TMEs by inducing metabolic reprogramming and attracting suppressive immune cells. Firstly, Arid5a enhances the suppressive effect of IDO against effector CD4^+^/CD8^+^ T-cells [379] by stabilizing *Ido1* mRNA and lowering intratumoral tryptophan levels (Figure 4). In tumor tissues, Ido1 expression enhances Treg differentiation and activation through the conversion of tryptophan to kynurenine, which activates aryl hydrocarbon receptors (AhRs) [380,381]. The activation of AhR leads to substantial gMDSC infiltration into the TME [382]. Secondly, Arid5a enhances the expression of the chemokine Ccl2 in the TME by stabilizing its mRNA and promoting the recruitment of immunosuppressive cells, such as Tregs and gMDSCs [377] (Figure 4). These findings highlight the diverse roles of Arid5a in the immune regulation and tumor progression, suggesting it as an attractive target for tumor immunotherapy and the treatment of inflammatory diseases.

## 9. Conclusions

With the remarkable advancements in epigenome analysis technology, driver mutations in cancer have been discovered, the classification of tumor cells and/or stromal cells by transcriptome analysis has been conducted, and the development of targeted drugs based on this information has progressed. This has led to the feeling of exhilaration that cancer may be conquered using the information from these studies. Nevertheless, subsequent research has demonstrated that, even when the effects of targeted drugs are substantial, cancer clones resistant to the targeted drugs eventually emerge, diminishing the belief that the targeted drugs by themselves can cure the disease. The main reasons contributing to the limited success of chemotherapies, such as FOLFIRINOX, and treatments targeting driver mutations and the cancer cell metabolism are the extensive diversity of mutant clones in tumor tissues and the acquisition of phenotypic plasticity owing to external effects from the TME.

On the other hand, immunotherapy based on the research achievements of Allison and Honjo, such as immune checkpoint inhibitors, has resulted in a paradigm shift in the development of new cancer treatments [383]. Although we were unable to cover it in this review, in recent years there has been substantial progress in the development of new tumor immunotherapy modalities, such as chimeric antigen receptor T-cell therapy, mRNA vaccines, etc. [384,385]. However, in PDAC, owing to the unique characteristics of its TME, namely, the physical and chemical barriers and the immunosuppressive biological environment, immunotherapy has not yet been applied.

Furthermore, many patients with PDAC present with local and distant metastases at the time of diagnosis, making it a challenging disease to treat. Current standard treatments add further selective pressure on cancer cells that have adopted mesenchymal characteristics, resulting in the generation of treatment-resistant tumor subclones and the induction of inflammatory responses that promote clonal evolution. Therefore, accumulating medical and biological insights into the fundamental mechanisms behind treatment resistance, the acquisition of invasive and metastatic traits, and the induction of immune evasion may provide numerous opportunities to further improve treatment outcomes. However, unless these mechanisms are understood systematically rather than as isolated processes, the results will be similar to those of traditional chemotherapy, targeted therapy, immunotherapy, and other treatments. It is crucial to deepen our understanding of the overall molecular mechanisms behind the mesenchymal plasticity involved in cancer malignancy, and to clarify the actual key factors and associated microenvironment mediators.

Recently, rapid advancements in single-cell and spatial technologies, together with advances in computational systems biology, have made it possible to investigate clonal dynamics evolution and gain insight into the roles of the TME at an unprecedented level of resolution and scale. Technologies for analyzing epigenetic markers, proteins, and metabolites at a single-cell resolution and high spatial accuracy are increasing [386,387]. By taking advantage of these technologies and integrating spatial and temporal information on the epigenome, metabolic systems, and immune systems from relevant preclinical and clinical samples, the underlying specificity of the process through which cancer acquires mesenchymal plasticity is expected to be further elucidated. With the progress of the above technologies, the key challenge in the single-cell era of cancer biology is to develop robust, reproducible, and transparent methods for analyzing, interpreting, and sharing the ever-growing “big data”. Education on algorithms and computational methodologies is necessary, together with the development of experimental strategies to rigorously test hypotheses based on tumor profiling, so to elucidate the molecular mechanisms involved in PDAC and their association with novel treatments. Therefore, to develop the next generation of cancer therapies, it is essential to foster interdisciplinary research efforts, specifically by strengthening the convergence of life sciences, physical sciences, engineering, computational sciences, and artificial intelligence.
